# Advances in Molecular Imaging for the Early Detection and Management of Alzheimer’s Disease

**DOI:** 10.7759/cureus.83127

**Published:** 2025-04-28

**Authors:** Shiv A Goel, Rishab Singh, Deepa Regina John, Pokhraj Suthar, Jagadeesh S Singh

**Affiliations:** 1 Department of Diagnostic Radiology and Nuclear Medicine, Rush University Medical Center, Chicago, USA; 2 Radiology, East Suffolk and North Essex Trust NHS, Colchester, GBR

**Keywords:** alzheimer’s disease, amyloid pet, early detection, mild cognitive impairment, molecular imaging, tau pet

## Abstract

Alzheimer's disease (AD) is a progressive neurodegenerative disorder characterized by impairments in memory and cognitive abilities. The development of new immunotherapies targeting beta-amyloid (Aβ) and tau protein deposition in the brain is ushering in great advances in clinical management. Advances in molecular imaging techniques, particularly positron emission tomography (PET) with amyloid and tau tracers, have facilitated the early detection of Alzheimer's pathology. We report a case of a 67-year-old patient presenting with mild cognitive impairment (MCI) who was confirmed to have AD on newer PET imaging. This report highlights the important role of molecular imaging in the early diagnosis of AD before significant clinical and functional decline sets in, thereby emphasizing its role in clinical practice.

## Introduction

With rising life expectancies, the prevalence of neurodegenerative diseases has surged globally. The World Health Organization predicts that dementia will affect 82 million individuals by 2030 [1]. Each year, approximately 10 million new cases are reported, with Alzheimer’s disease (AD) accounting for 60-70% of these cases [2-3]. Beyond the societal and economic burdens, AD directly impacts individuals and their families profoundly. It is a progressive neurodegenerative disorder characterized by impairments in memory and cognitive abilities. Deposition of intracerebral beta-amyloid (Aβ) and intracellular accumulation of tau tangles leading to synaptic dysfunction and death of neuronal cells have been implicated as the pathogenesis in AD. The pathophysiological process of AD has been shown to occur many years before the diagnosis and clinical presentation of AD dementia. This long preclinical phase of AD could provide a critical opportunity for therapeutic intervention, given the development of novel immunotherapies that target and reduce the amount of Aβ and tau protein deposition in the brain, thereby slowing the disease process.

Early and accurate diagnosis of AD is crucial for patient management, therapeutic intervention, and the development of disease-modifying treatments. Traditional diagnostic approaches, which rely on clinical assessment including neuropsychological testing, often identify AD at a relatively late stage when significant neuronal damage has already occurred. A paradigm shift has emerged, from a clinical diagnosis based only on symptoms and cognitive testing to one that is increasingly supported by biomarkers, namely amyloid and Tau positron emission tomography (PET) scan or CSF analysis, enabling the identification of Aβ or tau protein present early in the disease. Newer PET imaging uses radiolabeled tracers that bind selectively to amyloid and tau proteins, allowing for direct visualization and quantification of these pathologic protein deposits. CSF biomarkers reflect biochemical changes associated with AD pathology, providing an indirect measure of the presence of Aβ plaques and tau tangles. With continued advances in the AD biomarker research, the role of amyloid and tau PET imaging is becoming increasingly important for the early detection and management of AD.

## Case presentation

A 62-year-old right-handed female presented with complaints of progressive short-term memory loss over the past year. She reported difficulty recalling recent events, finding words, and remembering the location of personal items. She also had a history of mood fluctuations, increasing anxiety, sleep disturbances, decreased concentration, and episodes of agitation. There was no history of suicidal ideations or hallucinations. She had no history of smoking or significant alcohol consumption. Regarding family history, her father had been diagnosed with early-onset AD in his late 50s and passed away at 63 years.

**Figure 1:**
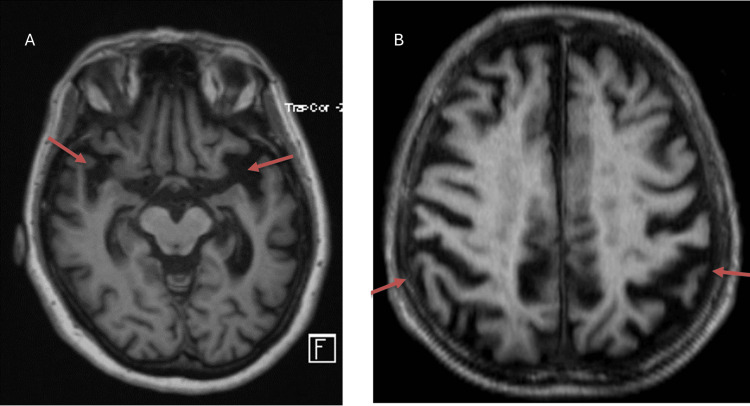
T1-weighted MRI axial images (A,B) showing cortical atrophy in the temporal and parietal regions (arrows) MRI: magnetic resonance imaging

On initial clinical evaluation, the patient had no complaints of tremors, seizures, facial asymmetry, speech difficulties, weakness, or headaches. On neuropsychological examination, she appeared nervous and anxious but showed no signs of hallucinations or psychosis. As for mental status examination, she was awake, alert, and oriented to time, place, and person. Her speech was fluent with intact comprehension, repetition, and naming abilities. She exhibited impaired attention and delayed recall, as evidenced by her Mini-Mental State Examination (MMSE) score of 26/30. Laboratory investigations, such as routine blood counts, metabolic profile, thyroid function tests, and Vit B12 levels, were normal. Routine CSF analysis showed normal cell count, glucose, and protein levels. Based on clinical findings of mild to moderate memory and cognitive impairment and given the family history of early-onset Alzheimer's in her father, there was clinical suspicion of Alzheimer's dementia. To confirm clinical findings and exclude other less likely etiologies like intracranial lesion, epilepsy, or other types of dementia, the patient underwent neuroimaging. Initial MRI showed mild cortical atrophy without evidence of intracranial lesions (Figure 1).

**Figure 2:**
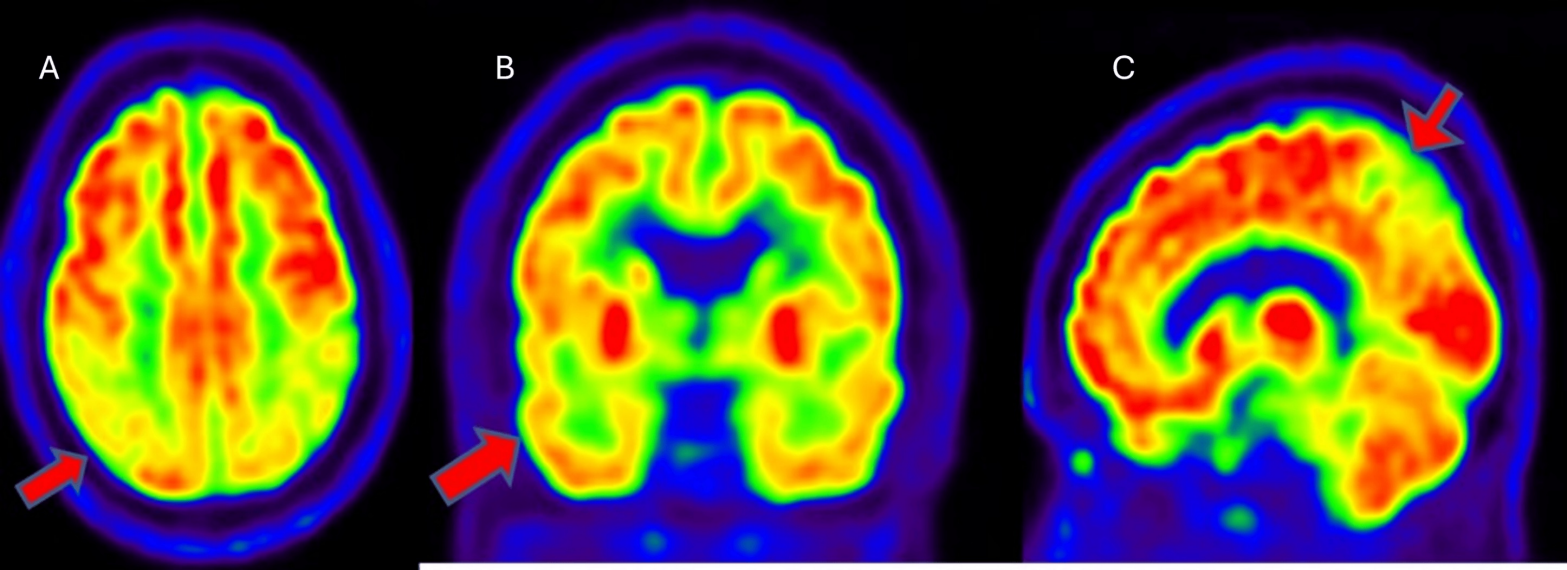
FDG-PET images (A: axial, B: coronal, C: sagittal) displaying hypometabolism in bilateral temporo-parietal lobes (red arrows) FDG-PET: fluorodeoxyglucose positron emission tomography

**Figure 3:**
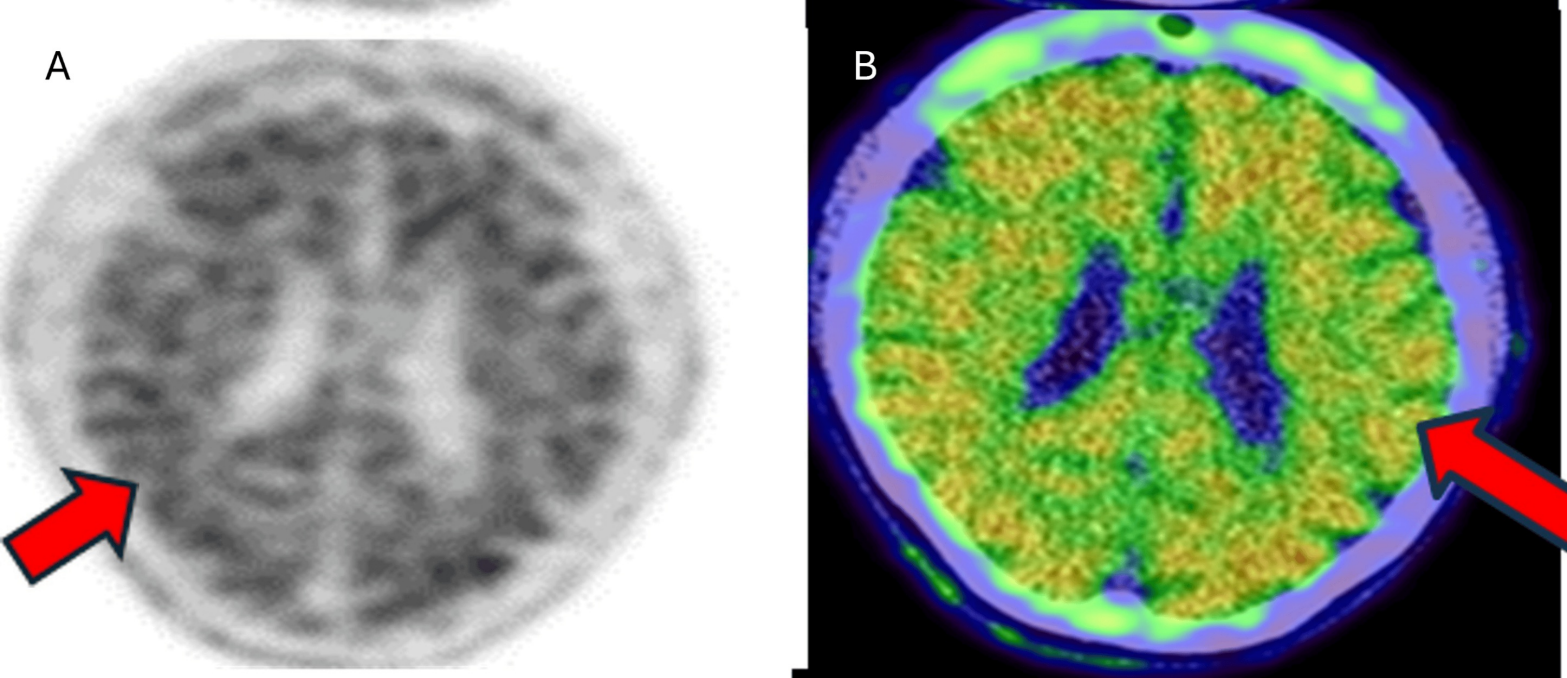
18F-Florbetapir axial PET images (A: grey scale, B: fused axial image) showing extensive beta-amyloid deposition in the cortical regions (grey matter) of the brain (red arrows) PET: positron emission tomography

**Figure 4:**
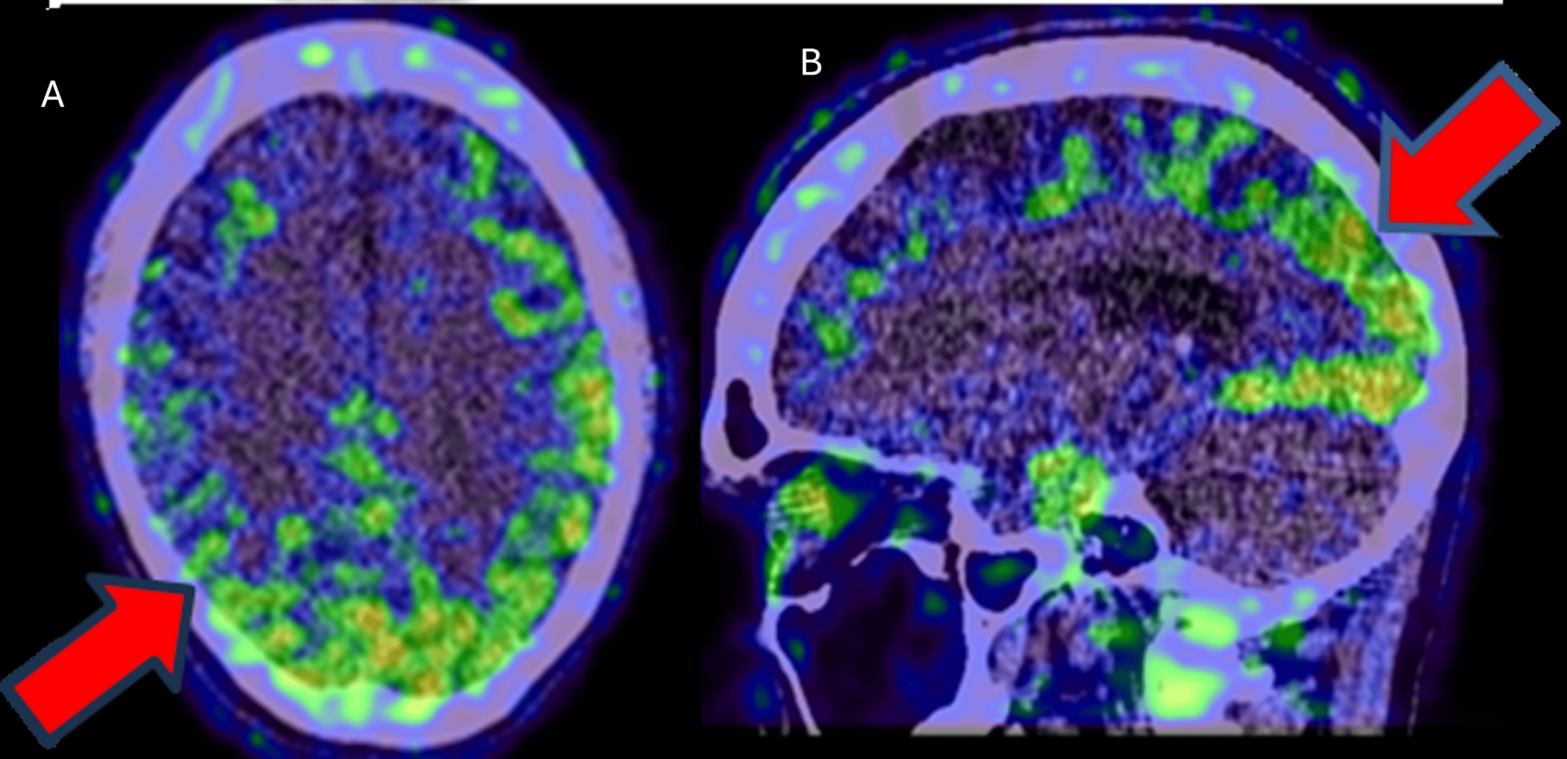
18F-GTP1 tau PET images (A: axial, B: sagittal fused images) illustrating tau protein accumulation in temporo-parietal regions (red arrows) PET: positron emission tomography

The patient subsequently underwent an F-18 FDG PET scan to confirm the type of dementia pattern. FDG PET showed hypometabolism in the bilateral temporal and parietal lobes, suggestive of Alzheimer's disease pattern (Figure 2). The patient was later referred to a University hospital where she was enrolled in research endeavors and underwent advanced PET imaging to detect the presence of beta-amyloid and tau protein. Amyloid PET (F-18 Florbetapir) demonstrated significant amyloid-beta deposition in the brain’s grey matter (Figure 3). Tau PET (F-18 GTP1) confirmed tau protein accumulation in the temporoparietal regions (Figure 4). Based on these findings, a diagnosis of AD was confirmed. The patient was enrolled in a clinical trial involving semorinemab, a monoclonal antibody targeting tau pathology. She was also started on cognitive rehabilitation therapy and lifestyle modifications, physical activity, and cognitive exercises.

Table 1 provides a summary of the patient's clinical findings and imaging results.

**Table 1:** Summary of clinical findings and imaging results CBC: complete blood count; CSF: cerebrospinal fluid; FDG-PET: fluorodeoxyglucose positron emission tomography; MRI: magnetic resonance imaging; TSH: thyroid-stimulating hormone

Clinical findings and imaging results	
Clinical finding	Result/observation
Patient age, years	62
Diagnosis	Alzheimer’s disease
Presenting symptoms	Progressive short-term memory loss, difficulty in recalling recent events, finding words, and remembering the location of personal items
Family history	Early-onset Alzheimer’s disease in the father
Lab findings	CBC, TSH, vitamin B12, and CSF analysis were unremarkable
Surgical procedures	none
Imaging	Observations
MRI (brain)	Cortical atrophy without evidence of intracranial lesions (Figure 1)
F18-FDG PET scan	Hypometabolism in the bilateral temporal and parietal lobes (Figure 2)
Amyloid PET (F-18 Florbetapir)	Positive amyloid-beta deposition in the brain’s grey matter (Figure 3)
Tau PET (F-18 GTP1)	Positive tau protein accumulation in the temporoparietal regions (Figure 4)
Current referral	Clinical trials exploring semorinemab, a monoclonal antibody targeting tau pathology

## Discussion

AD primarily affects individuals aged 65 and older, with early-onset cases occurring in less than 10% of patients. Symptoms, including memory impairment and cognitive decline, often begin more than a decade before clinical diagnosis. Early-onset AD has been linked to genetic mutations, such as those involving the APOE gene. The pathophysiology involves extracellular amyloid-beta plaques and intracellular tau tangles, leading to synaptic dysfunction and neuronal death [4,5,6]. Early and accurate diagnosis of AD is crucial for patient management and therapeutic intervention. Traditional diagnostic approaches, relying on clinical assessment including neuropsychological testing, often identify AD at a relatively late stage when significant neuronal damage has already occurred. Moreover, neuropathological studies show that amyloid plaques and neurofibrillary tangles are found in only about 85% of cases with a clinical diagnosis of AD dementia, highlighting the limited specificity of clinical criteria in detecting AD neuropathology [7]. Hence, there is an increasing emphasis on the clinical use of biomarkers to facilitate early and specific diagnosis of AD.

Two biomarker modalities are now well-validated and approved components in the diagnostic workup of AD patients in clinical settings. Advanced amyloid and tau PET imaging and CSF biomarkers enable in vivo detection of cerebral amyloid and tau pathology and provide critical insights into the underlying disease mechanisms, aiding in the identification of AD pathological changes even at the preclinical or mild cognitive impairment (MCI) stages of AD [8]. Amyloid and Tau PET imaging uses novel radiolabeled tracers that bind selectively to amyloid and tau proteins, allowing for direct visualization and quantification of these pathologic protein deposits, previously only possible through histopathological examination. When combined with advanced MRI techniques and CSF biomarkers, these modalities enhance diagnostic accuracy and enable tailored interventions in the prodromal stage of the disease [9,10]. MRI with diffusion tensor imaging can reveal structural changes, such as hippocampal and cortical atrophy, although these changes appear later in disease progression. Diffusion tensor imaging can identify white matter microstructural changes. FDG-PET detects hypometabolism in specific brain regions, aiding in differentiating AD from other dementias. Amyloid PET provides in vivo visualization of amyloid plaque deposition, a hallmark of AD. Tau PET detects tau tangles, critical for staging disease progression.

The integration of amyloid and tau imaging into clinical practice is facilitated by the "A/T/N" biomarker classification system [11-12]. In the proposed A/T/N classification system, the AD biomarkers are divided into three binary classes: “A” refers to the value of an Aβ biomarker (amyloid PET or CSF Aβ42); “T,” the value of a tau pathology biomarker (CSF p-tau or tau PET); and “N,” a quantitative or topographic biomarker of neurodegeneration or neuronal injury (CSF t-tau, FDG-PET, or structural MRI). Advanced PET imaging and CSF biomarkers are now established tools in the diagnostic workup of AD patients, and their use is anticipated to increase with the introduction of new disease-modifying therapies.

Although these biomarkers are comparable alternatives in research settings to determine amyloid status, biomarker testing in clinical practice requires careful consideration of the strengths and limitations of each modality, as well as the specific clinical context, to identify which test is best suited for each patient. Current literature suggests that both advanced PET imaging and CSF biomarkers will continue to play an increasingly important role in the diagnostic workup of patients with AD. The best PET measures performed for the presence of beta-amyloid deposition in the brain showed a sensitivity of 88-91% and specificity of 80-84%. The best CSF measures for identifying patients with MCI and suspected AD (MCI-AD) were CSF measurement of Aβ42/total tau (t-tau) and Aβ42/hyperphosphorylated tau (p-tau) ratios, with a sensitivity of 94-97% and a specificity of 83-85% [13-15].

These two biomarkers have also played a pivotal role in the design and implementation of clinical trials for AD. These biomarkers have been successfully used to select participants for clinical trials using disease-modifying drugs, including the approved drugs aducanumab and lecanemab, allowing for the exclusion of those patients with clinical AD but who do not have biomarker evidence for Aβ pathology, and thus would not benefit from treatment [16]. However, in clinical settings, amyloid PET quantification has been well validated and helps in assessing amyloid deposition clearance and in supporting treatment discontinuation decisions. In summary, advanced amyloid and tau PET imaging, along with CSF biomarkers, allow clinicians to identify AD in its preclinical stage, enabling earlier interventions with immunotherapies and more precise management strategies [17-19].

## Conclusions

AD is a progressive neurodegenerative disease associated with substantial personal and societal challenges. Early detection of AD through advanced molecular imaging, such as amyloid and tau PET along with CSF biomarkers, represents a paradigm shift in its diagnosis and treatment. With continued advances in the AD biomarker research and increasing availability of disease-modifying immunotherapies, these molecular imaging modalities will play an essential role in early diagnosis, patient selection, and timely therapeutic intervention.
